# Preventing the adverse cardiovascular consequences of allogeneic stem cell transplantation with a multi-faceted exercise intervention: the ALLO-Active trial protocol

**DOI:** 10.1186/s12885-022-09793-w

**Published:** 2022-08-17

**Authors:** Hayley T. Dillon, Nicholas J. Saner, Tegan Ilsley, David Kliman, Andrew Spencer, Sharon Avery, David W. Dunstan, Robin M. Daly, Steve F. Fraser, Neville Owen, Brigid M. Lynch, Bronwyn A. Kingwell, Andre La Gerche, Erin J. Howden

**Affiliations:** 1 Baker Heart and Diabetes Institute, Melbourne, Australia; 2 Institute for Physical Activity and Nutrition, School of Exercise and Nutrition Sciences, Deakin University, Geelong, Australia; 3 Central Clinical School, Faculty of Medicine, Nursing and Health Sciences, Monash University, Melbourne, Australia; 4 Malignant Haematology and Stem Cell Transplantation Service, Alfred Hospital, Melbourne, Australia; 5 Mary MacKillop Institute for Health Research, Australian Catholic University, Melbourne, Australia; 6 Centre for Urban Transitions, Swinburne University of Technology, Melbourne, Australia; 7 Cancer Epidemiology Division, Cancer Council Victoria, Melbourne, Australia; 8 Centre for Epidemiology and Biostatistics, Melbourne School of Population and Global Health, University of Melbourne, Parkville, Australia; 9 CSL Ltd, Melbourne, Australia

**Keywords:** Exercise training, Allogeneic Stem Cell Transplant, Cardiovascular Health

## Abstract

**Background:**

Allogeneic stem cell transplantation (allo-SCT) is a potentially lifesaving treatment for high-risk hematological malignancy, but survivors experience markedly elevated rates of cardiovascular disease and associated functional impairment. Mounting evidence suggests regular exercise, combined with a reduction in sedentary time through replacement with light exercise may be a useful therapeutic strategy for the prevention of cardiovascular comorbidities. However, this type of intervention has yet to be evaluated in patients undergoing allo-SCT. The ALLO-Active study will evaluate the efficacy of a ~ 4 month multi-faceted exercise intervention, commenced upon admission for allo-SCT, to preserve peak oxygen uptake (VO_2_peak) and peak cardiac output, compared with usual care. The study will also evaluate the effect of the intervention on functional independence, quality of life, and symptoms of fatigue.

**Methods:**

Sixty adults with hematological malignancy scheduled for allo-SCT will be randomly assigned to usual care (*n* = 30) or the exercise and sedentary behaviour intervention (*n* = 30). Participants assigned to the intervention will complete a thrice weekly aerobic and progressive resistance training program and concomitantly aim to reduce daily sedentary time by 30 min with short, frequent, light-intensity exercise bouts. Participants will undergo testing prior to, immediately after inpatient discharge, and 12 weeks after discharge. To address aim 1, VO_2_peak and peak cardiac output (multiple primary outcomes, *p* < 0.025) will be assessed via cardiopulmonary exercise testing and exercise cardiac magnetic resonance imaging, respectively. Secondary outcomes include functional independence (defined as VO_2_peak ≥ 18.mL.kg^−1^.min^−1^), quality of life, and fatigue (assessed via validated questionnaire). Exploratory outcomes will include indices of resting cardiac, vascular, and skeletal muscle structure and function, cardiovascular biomarkers, anxiety and depression, transplant outcomes (e.g., engraftment, graft-versus-host disease), and habitual physical activity, sedentary time, and sleep.

**Discussion:**

Multi-faceted exercise programs are a promising approach for ameliorating the cardiovascular consequences of allo-SCT. If this intervention proves to be effective, it will contribute to the development of evidence-based exercise guidelines for patients undergoing allo-SCT and assist with optimising the balance between acute cancer management and long-term health.

**Trial Registration:**

Australian New Zealand Clinical Trials Registry (ANZCTR), ID: 12619000741189. Registered 17 May 2019.

## Background

Since its inception in 1957, allogeneic stem cell transplantation (allo-SCT) has developed from being an experimental procedure to being the best chance of cure for many high-risk hematological malignancies. Accordingly, the last decade has seen a ~ two-fold increase in the number of allo-SCTs performed globally, and the 5-year overall and progression free survival rates are approaching 62% and 52%, respectively, up from 46% and 42% during 1980–1989 [1, 2]. However, the quality and longevity of survival continues to be compromised by treatment-related cardiovascular adverse effects [3]. Owing to the synergistic effects of toxic anti-cancer therapies [4], prolonged bedrest [5], and the inflammatory perturbations of allografting [6, 7], allo-SCT survivors are susceptible to multi-organ impairment. This has resulted in a population characterised by fatigue [8], poor quality of life [9], reduced muscular strength [10], low aerobic capacity (peak oxygen uptake [VO_2_peak]) [11, 12], and an associated 2-to-fourfold elevation in cardiovascular morbidity and mortality [6, 13–18]. Indeed, in a period of 4–12 weeks (inpatient stay and acute outpatient recovery), allo-SCT recipients experience declines in VO_2_peak approximating the degree of cardiovascular aging typically expected over 2–3 decades [11, 12]. Moreover, as many as 53% of allo-SCT survivors meet criteria for functional disability (VO_2_peak < 18 ml.kg^−1^.min^−1^) [12, 19]—a clinical threshold associated with poorer quality of life and a 7-to-ninefold increased risk of heart failure and all-cause mortality [12, 20]. As such, there is growing interest in therapeutic strategies to reduce cardiovascular risk and improve quality of life in allo-SCT survivors.

Current approaches for mitigating cardiovascular morbidity in patients undergoing allo-SCT include modifications in transplant conditioning intensity and cardiovascular pharmacotherapy. Reduced-intensity transplant conditioning is associated with less cardiac toxicity, although at the cost of higher relapse and graft-versus-host disease (GVHD) rates [21, 22]. Similarly, prophylactic cardioprotective pharmacotherapy can reduce the risk of treatment-related cardiac dysfunction [23], but is not routinely recommended for allo-SCT patients due to concerns surrounding polypharmacy (e.g., increased healthcare costs, adverse drug events and/or drug interactions), overall therapeutic efficacy, and the reality that many patients would be treated unnecessarily [24, 25]. In addition, cardiac-specific pharmacotherapies fail to address impairments in the non-cardiac, ‘peripheral’ (hematological, vascular, skeletal muscle) components of the oxygen transport cascade, which also contribute to the decline in VO_2_peak and cardiovascular health among allo-SCT recipients [26–30].

Combined aerobic and resistance exercise has emerged as a promising preventative therapy for cancer treatment-related cardiovascular dysfunction and functional impairment due to its ability to target the entire spectrum of the oxygen transport cascade. Further, resistance training is an effective tool for stimulating muscle hypertrophy and improving strength—both of which are profoundly impaired among allo-SCT survivors and have been shown to predict poor clinical outcomes [10]. In the context of allo-SCT, aerobic and resistance exercise training has shown promise in improving fatigue [31–34] and quality of life [31, 35, 36], but its efficacy in preserving cardiovascular function is yet to be fully investigated. Indeed, previous randomised controlled trials in allo-SCT recipients have demonstrated favourable effects of combined aerobic and resistance training on indices of aerobic capacity [11, 31, 33, 35–39]—a marker of integrative cardiovascular function strongly associated with cardiovascular and all-cause mortality [19, 40, 41]. However, these trials have relied on indirect estimates of VO_2_peak, with many commencing after the intensive inpatient treatment period wherein cardiovascular insults are arguably greatest, and hence, irreversible cardiovascular impairment may have already occurred. Moreover, the degree to which the beneficial effects on VO_2_peak are mediated by central (cardiac) versus peripheral (hematological, vascular, skeletal muscle) factors remains unknown and is integral to establishing the therapeutic utility of exercise in preventing allo-SCT-induced cardiovascular dysfunction. And finally, none of these trials employed the use of high-intensity interval-based training which has been shown to stimulate a significantly higher improvement in VO_2_peak and overall cardiovascular risk profile compared to moderate-intensity continuous training [42]. Therefore, there is a need for randomised controlled trials—commencing early in the transplant process and including comprehensive cardiovascular evaluation—to determine the efficacy of combined aerobic (moderate-intensity continuous and high-intensity interval) and resistance exercise in preserving the cardiovascular health and functional status of patients undergoing allo-SCT.

Beyond the promotion of purposeful exercise, the reduction and interruption of prolonged periods of time spent sitting or reclining has emerged as an important health target. This is based on the evidence that time spent sedentary poses risks to cardiovascular health that are additional to those of lack of physical activity [43–45]. In the context of allo-SCT, the necessary confinement to a hospital room (~ 3–5 weeks) generally coincides with a marked increase in what is considered an extreme form of sedentary behaviour—bedrest. Thus, a multi-faceted intervention that aims to both increase purposeful exercise and interrupt prolonged periods of sedentary time through replacement with light exercise might be the ideal therapeutic tool to counteract the negative cardiovascular consequences associated with physical deconditioning, cancer therapies, and the pathological perturbations of allografting. Indeed, evidence suggests that structured aerobic and resistance exercise, combined with a reduction in sedentary time, has the potential to have widespread effects on important cardiovascular organs including the heart [46–49], skeletal muscle [50–54], vasculature [47, 55, 56], and blood cells [49], as well as exerting systemic effects that favourably impact these organs [52, 57–60]. No studies have investigated the efficacy of a multi-faceted exercise intervention in preserving cardiovascular function, improving quality of life, and reducing symptoms of fatigue in patients undergoing allo-SCT.

Therefore, in a two-arm randomised controlled trial, the ALLO-Active study will evaluate the efficacy of a ~ 4 month multi-faceted exercise intervention—commenced upon admission for allo-SCT—in preserving cardiovascular function (assessed as VO_2_peak and peak exercise cardiac output; primary endpoints) and improving quality of life, fatigue, and functional independence (VO_2_peak ≥ 18 ml.kg^−1^.min^−1^; secondary endpoints) compared to a usual clinical care control. The intervention is designed to increase purposeful aerobic and resistance exercise and reduce sedentary time through replacement with light exercise and we hypothesise that it will:

1. Attenuate the decline in VO_2_peak and peak exercise cardiac output as compared with usual care.

2. Improve quality of life, reduce symptoms of fatigue, and improve functional independence compared with usual care.

Exploratory aims include assessing the effect of the intervention on additional *cardiac* factors such as cardiac reserve, cardiac biomarkers, and indices of resting cardiac structure and function, as well as *peripheral* factors including vascular compliance, body composition, indices of skeletal muscle structure and function, metabolic biomarkers, and habitual physical activity, sedentary behaviour, and sleep. This study will also explore the intervention's effect on anxiety and depression and transplant-related variables such as engraftment, immune reconstitution, and GVHD.

## Methods

### Study design

**Fig. 1:**
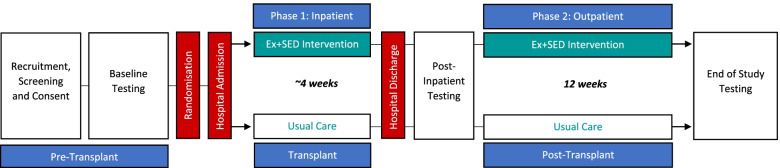
Overview of the ALLO-Active trial design. A comprehensive battery of tests will be conducted in both groups during pre-transplant (baseline) and end of study testing (12-week post-discharge), while an abbreviated version of testing will be performed at post-inpatient testing (detailed in Table 3)

The ALLO-Active trial is a two-arm parallel group, randomised controlled trial in patients with hematological malignancy scheduled to undergo allo-SCT. The purpose of this study is to determine the efficacy of an innovative, individualised multi-faceted exercise intervention delivered during allo-SCT and acute recovery (Phase 1—Inpatient) and the 12 weeks post hospital discharge (Phase 2—Outpatient), compared against a usual clinical care control group (Fig. 1). The study will be conducted at the Baker Heart and Diabetes Institute (BHDI) and Alfred Hospital in Melbourne, Victoria, Australia, and a total of 60 patients will be recruited and randomly allocated to either the exercise & sedentary behaviour (Ex + SED) or Usual Care (UC) arm of the study (*n* = 30 in each). Study assessments will be performed at the BHDI at baseline (~ 2 weeks prior to hospital admission), post-inpatient admission (~ 1 week after inpatient discharge), with final testing to be completed 12 weeks following discharge from inpatient stay.

### Ethics

The ALLO-Active trial is registered with the Australian New Zealand Clinical Trials Registry (ANZCTR; registration number: ACTRN12619000741189), funding has been provided via a World Cancer Research Fund International (WCRF) grant (WCRFI 2019–1666) and the protocol has been approved by the Alfred Hospital Human Research Ethics Committee (project number: 134/19). The study team will communicate any important protocol modifications to the trial registry and the Alfred Hospital Human Research Ethics Committee.

### Randomisation

Following completion of baseline testing, participants will be randomly allocated (1:1 ratio) to either the UC or Ex + SED group by an independent researcher via a computer-generated random number sequence. Stratified permuted blocked randomisation will be used with participants stratified by transplant conditioning intensity (myeloablative or reduced intensity conditioning) and sex (male or female). Information relating to the allocation sequence will be kept on a separate, password protected database accessible only to the independent researcher and principle investigator.

### Blinding

Given the nature of the intervention, it will not be possible to blind participants to their group assignment. The analysis of all outcome measures (e.g., cardiopulmonary exercise test data, echocardiography, cardiac magnetic resonance imaging etc.) will be performed by researchers blinded to participant group assignment and baseline testing values.

### Participants and recruitment

Participants scheduled to undergo allo-SCT at the Alfred Hospital, Melbourne, Victoria will undergo initial eligibility screening by their nurse transplant coordinator. If identified as interested and eligible based on the criteria outlined in Table 1, the nurse transplant coordinator will provide the participant’s contact details to the ALLO-Active study coordinator. Following further discussion of the purpose of the study and participation requirements via telephone with the ALLO-Active study coordinator, the participant will be provided a participant information and consent form.

**Table 1:** Participant eligibility criteria

Inclusion Criteria	Exclusion Criteria
- Age ≥ 18 years	- Unable to communicate or read in English
- Scheduled for allo-SCT	- Contraindications to 3 T MRI (e.g., pacemaker or implanted metallic device)
- Able to exercise at time of enrolment	

After informed consent is acquired, participant demographics, health status, current medications, and cardiovascular risk profile (including family history of CVD, presence of cardiovascular risk factors or overt cardiovascular disease, smoking status, alcohol consumption and menstrual reproductive history) will be documented by questionnaire at baseline. Additional relevant medical history will be extracted from medical records and will include information related to each participants hematological cancer diagnosis, prior anti-cancer treatment history, stem cell transplant conditioning regimen (protocol and dosage [intensity and agents]) and donor (relation, graft source, HLA matching).

### Intervention

This is an individualised, multi-faceted intervention designed to increase aerobic and resistance exercise and reduce prolonged sedentary behaviour with the global aim of attenuating the negative impact of allo-SCT on cardiovascular function. The two intervention components will be delivered in parallel, over two distinct phases: Phase 1—commenced upon inpatient admission for allo-SCT and continued for the duration of inpatient care (~ 4 weeks); and Phase 2—commenced immediately post-discharge from inpatient care and continued for 12 weeks. Participants will be provided with a wrist worn smartwatch (Fitbit Ionic or Fitbit Versa 2, Fitbit, Inc., San Francisco, CA, USA) that will be worn at all times to facilitate and monitor adherence to the intervention. Each device will be connected to a cloud-based data aggregation platform (Fitabase Small Steps Labs, LLC, San Diego, CA) wherein 24/7 heart rate, step count, and intensity (time spent in light, moderate and high intensity activity) data will be captured in real-time. An overview of the exercise intervention can be found in Table 2.

**Table 2:** Outline of the ALLO-Active multi-faceted Ex + SED program

Phase	Weeks	Session Type	Frequency (per/week)	Duration/Dose	Intensity
1—Inpatient	1, 3–4	Continuous Aerobic & Resistance	2	*AT:* 20 min *RT:* 4–6 ex, 1 set × 15 reps	*AT:* 60% VO_2_peak, 12–14 RPE *RT:* 14–16 RPE
		Interval & Resistance	1	*AT:* 4 × 2 min on, 3 min off *RT:* 4–6 ex, 1 set × 15 reps	*AT:* 80% VO_2_peak, 85–95% HRpeak, and/or ≥ 17 RPE *RT:* 14–16 RPE
	2	Continuous Aerobic & Resistance	3	*AT:* 20 min *RT:* 4-6 ex, 1 set × 15 reps	*AT:* 60% VO_2_peak, 12–14 RPE *RT:* 14–16 RPE
	1–4	Sedentary Behaviour Breaks	7	10 x ≥ 3 min per day, completed hourly	Light (normal breathing rate – can sing or talk)
2—Outpatient	1–2	Continuous Aerobic & Resistance	1	*AT:* 20 min (wk1), 21 min (wk2) *RT:* 6–8 ex, 2 sets × 10–15 reps	*AT:* 60% VO_2_peak, 12–14 RPE *RT:* 14–16 RPE
		Interval & Resistance	1	*AT:* 4 × 2 min on, 3 min off *RT:* 6–8 ex, 2 sets × 10–15 reps	*AT:* 80% VO_2_peak, 85–95% HRpeak, and/or ≥ 17 RPE *RT:* 14–16 RPE
		Continuous Aerobic (Home)	1	*AT:* 20 min (wk1), 21 min (wk2)	*AT:* 60% VO_2_peak, 12–14 RPE
	3–4	Continuous Aerobic & Resistance	1	*AT:* 22 min (wk3), 23 min (wk4) *RT:* 6–8 ex, 2 sets × 10–15 reps	*AT:* 60% VO_2_peak, 12–14 RPE *RT:* 14–16 RPE
		Interval & Resistance	1	*AT:* 6 × 2 min on, 3 min off *RT:* 6–8 ex, 2 sets × 10–15 reps	*AT:* 80% VO_2_peak, 85–95% HRpeak, and/or ≥ 17 RPE *RT:* 14–16 RPE
		Continuous Aerobic (Home)	1	*AT:* 22 min (wk3), 23 min (wk4)	*AT:* 60% VO_2_peak, 12–14 RPE
	5–6	Continuous Aerobic & Resistance	1	*AT:* 24 min (wk5), 25 min (wk6) *RT:* 6–8 ex, 2 sets × 10–15 reps	*AT:* 60% VO_2_peak, 12–14 RPE *RT:* 14–16 RPE
		Interval & Resistance	1	*AT:* 4 × 3 min on, 3 min off *RT:* 6–8 ex, 2 sets × 10–15 reps	*AT:* 80% VO_2_peak, 85–95% HRpeak, and/or ≥ 17 RPE *RT:* 14–16 RPE
		Continuous Aerobic (Home)	1	*AT:* 24 min (wk5), 25 min (wk6)	*AT:* 60% VO_2_peak, 12–14 RPE
	7–8	Continuous Aerobic & Resistance	1	*AT:* 25 min (wk7), 26 min (wk8) *RT:* 6–8 ex, 2 sets × 10–15 reps	*AT:* 60% VO_2_peak, 12–14 RPE *RT:* 14–16 RPE
		Interval & Resistance	1	*AT:* 4 × 3 min on, 2 min off *RT:* 6–8 ex, 2 sets × 10–15 reps	*AT:* 80% VO_2_peak, 85–95% HRpeak, and/or ≥ 17 RPE *RT:* 14–16 RPE
		Continuous Aerobic (Home)	1	*AT:* 25 min (wk7), 26 min (wk8)	*AT:* 60% VO_2_peak, 12–14 RPE
	9–10	Continuous Aerobic & Resistance	1	*AT:* 27 min (wk9), 28 min (wk10) *RT:* 6–8 ex, 2 sets × 10–15 reps	*AT:* 60% VO_2_peak, 12–14 RPE *RT:* 14–16 RPE
		Interval & Resistance	1	*AT:* 5 × 3 min on, 2 min off *RT:* 6–8 ex, 2 sets × 10–15 reps	*AT:* 80% VO_2_peak, 85–95% HRpeak, and/or ≥ 17 RPE *RT:* 14–16 RPE
		Continuous Aerobic (Home)	1	*AT:* 27 min (wk9), 28 min (wk10)	*AT:* 60% VO_2_peak, 12–14 RPE
	11–12	Continuous Aerobic & Resistance	1	*AT:* 29 min (wk11), 30 min (wk12) *RT:* 6–8 ex, 2 sets × 10–15 reps	*AT:* 60% VO_2_peak, 12–14 RPE *RT:* 14–16 RPE
		Interval & Resistance	1	*AT:* 6 × 3 min on, 2 min off *RT:* 6–8 ex, 2 sets × 10–15 reps	*AT:* 80% VO_2_peak, 85–95% HRpeak, and/or ≥ 17 RPE *RT:* 14–16 RPE
		Continuous Aerobic (Home)	1	*AT:* 29 min (wk11), 30 min (wk12)	*AT:* 60% VO_2_peak, 12–14 RPE
	1–12	Sedentary Behaviour Breaks	7	10 x ≥ 3 min per day, completed hourly	Light (normal breathing rate – can sing or talk)

Abbreviations: *AT* Aerobic training, *Ex* Exercise, *RPE* Rating of perceived exertion, *RT* Resistance training

#### Phase 1 (inpatient phase, ~4 weeks)

The individualised, structured, exercise training program delivered during allo-SCT, and acute inpatient recovery will consist of three, 30–45 min sessions of supervised, combined aerobic and resistance exercise per week. Each participant will receive their own stationary exercise bike and set of therabands. Sessions will be conducted within the patient’s room on the hematology oncology ward of the Alfred Hospital, and under the supervision of a physiotherapist, or exercise physiologist.

##### Structured aerobic exercise training

The structured aerobic component of the program will comprise both continuous moderate-intensity exercise and high-intensity interval-based exercise training. Interval training sessions will be performed on an upright cycle ergometer (placed within the patient’s hospital room), while continuous sessions will be performed on an upright cycle ergometer or, when warranted, as a walk around the hematology oncology ward. Continuous moderate-intensity aerobic exercise will be completed twice-weekly, for 20 min, at a workload and/or heart rate corresponding to ~ 60% VO_2_peak or rating of perceived exertion (RPE) 12–14 on the 20-point RPE scale. High-intensity interval training will be completed once weekly (except for the week following stem cell transplantation) and consist of 4 × 2 min intervals at a workload corresponding to ≥ 80% VO_2_peak, 85–95% HR_peak_ and/or RPE ≥ 17, interspersed with 3 min of light intensity cycling for active recovery. All sessions will include a 3–5 min aerobic warm-up and cool-down. Subjective and objective exercise intensity will be monitored using Borg’s 6–20-point RPE scale and participant’s Fitbit heart rate monitor, respectively, and used to adjust exercise workloads to account for the undulating health status inherent in patients undergoing allo-SCT.

##### Structured resistance exercise training

The progressive resistance training component will be completed immediately following the aerobic component and comprise 4–6 compound, body weight and resistance band style exercises with the goal of maintaining muscle strength and mass. The moderate intensity resistance exercise program will include 2 lower body exercises (e.g., squats, lunges, glute bridge, step-ups) and 2 to 4 upper body exercises incorporating both pulling (e.g., resistance band rows) and pushing (e.g., incline push-ups, resistance band chest press) actions to address all major muscle groups of the body. Participants will perform each exercise for 1 set of 15 repetitions at 14–16 RPE and in a slow, controlled manner. The resistance will be increased, or exercise progressed, once 15 repetitions can be completed at ≤ 13 RPE.

##### Sedentary time reduction

Alongside and independent of the thrice-weekly exercise program, participants will receive 10 daily prompts (at hourly intervals) via their Fitbit smartwatch to interrupt their sedentary time with ≥ 3 min of light intensity exercise with the overall objective of reducing total sedentary time by ≥ 30 min/day. When prompted, participants will have the option to break up their sedentary time with ≥ 3 min of light walking, light cycling, or with a simple body-weight resistance exercise circuit comprising squats, calf raises and gluteal kickbacks (each exercise performed for 20 s each, repeated 3 times). When limited to bed rest, participants will be instructed to modify the body-weight resistance exercise circuit to be completed in a supine position in bed. Participants will utilise the exercise tracking feature on their Fitbit to record each sedentary behaviour break.

#### Phase 2 (outpatient phase, 12 weeks)

In phase 2, the individualised exercise program will continue, but with an increase in training volume to 45–60 min per session, and a reduced frequency of in-person supervision to twice per week. Two supervised exercise sessions will be conducted at the BHDI and comprise both an aerobic and resistance training component. The third session will be unsupervised, be aerobic in nature and conducted at each participant’s home. The outpatient program is designed to facilitate a progressive increase in exercise volume of 5–10% each week in line with American College of Sports Medicine (ACSM) guidelines [61].

##### Structured aerobic exercise training

During phase 2, participants will continue to complete one high-intensity interval (supervised) and two continuous moderate-intensity based aerobic exercise sessions per week (one supervised, one unsupervised), but with a progressive increase in volume over the 12 weeks. As per phase 2, continuous moderate-intensity sessions will be performed at a workload and/or heart rate corresponding to ~ 60% VO_2_peak and/or RPE 12–14, beginning at 20 min in week one but progressing one minute each week until participants are completing a total of 30 min in week 12. Intervals will be performed at a workload corresponding to ≥ VO_2_peak, 85–95%HR_peak_ and/or RPE ≥ 17, beginning with 4 × 2 min intervals, interspersed with 3 min active recovery, but gradually progressing to 6 × 3 min intervals, interspersed with 2 min of active recovery. Supervised interval and aerobic training will be performed on an upright cycle ergometer or treadmill, while the unsupervised home-based continuous session will be completed in a mode of the participant’s choice (walking or cycling).

##### Structured resistance exercise training

During phase 2, progressive resistance training will be completed twice weekly, with an increase in total volume and intensity from phase 1. Participants will complete six to eight compound, body-weight, free weight and/or machine-based exercises (3–4 lower body, 3–4 upper body) with the goal of increasing whole body muscle strength and mass. Example exercises include leg press, squats, deadlifts, lunges, and step-ups for lower body, and latissimus dorsi pull-down, seated horizontal row, chest press, and overhead press for upper body. Each exercise will be performed for 2 sets of 10–15 repetitions at RPE 14–16 with 1–2 min rest in between each set. Progressive overload will be applied via periodised augmentation of repetitions and resistance. Specifically, repetitions will be increased by 1–2 each week until participants can exceed the upper end of the desired repetition range (e.g., 15 repetitions), or perform the prescribed repetitions at RPE ≤ 13, after which, the resistance will be increased by 5–10% and participants will resume at a lower repetition goal before progressively increasing again.

##### Sedentary time reduction program

As per phase 1, participants will continue to interrupt their sitting time with 10 daily, hourly light activity exercise breaks.

### Safety precautions: relative and absolute contraindications

Individuals undergoing allo-SCT experience varying degrees of pancytopenia and a range of complications including, but not limited to, loss of appetite, nausea, infections, GVHD, and hemodynamic instability. The prescription of the pre-stated exercise intervention will therefore be individually modified in accordance with patient health status in consultation with the treating hematologist. Absolute contraindications to exercise will include febrility (body temperature > 38 °C), severe pain, nausea and dizziness, platelet count < 15 × 10^9^/L, hemoglobin count < 75 g/L, resting systolic blood pressure > 200 mmHg or < 90 mmHg, resting diastolic blood pressure > 110 mmHg or < 60 mmHg, or resting heart rate > 120 beats/min. Relative contraindications to exercise will include platelet count ≥ 15 × 10^9^/L but < 20 × 10^9^/L, hemoglobin count ≥ 75 g/L but < 80 g/L, resting systolic blood pressure 160–200 mmHg or 90–100 mmHg, resting diastolic blood pressure 90–110 mmHg and resting heart rate 100–120 beats/min. In the instance of a relative contraindication, the prescribed exercise intensity will be limited to light-to-moderate, and signs and symptoms of tachycardia, hypotension and/or hypertension will be closely monitored. Under all circumstances, if a patient becomes symptomatic during activity (e.g., pain, dizziness, nausea), the exercise session will be ceased.

### Usual care control group

Participants randomised to usual care will receive usual medical care throughout the trial. Usual care participants will be fitted with wrist-worn Fitbit activity trackers which will be connected to the cloud-based data aggregation platform (Fitabase Small Steps Labs, LLC, San Diego, CA) to measure day-to-day activity but will not receive exercise support. Additionally, to maintain study engagement, to reduce social-interaction mediated bias on quality of life and mood outcomes, and for equipoise, the study coordinator will visit usual care participants in their hospital room (Phase 1—Inpatient) and at the hematological oncology centre (Phase 2—Outpatient) on a twice weekly basis.

### Experimental protocol

The experimental measures, and their timing are summarised in Table 3. In accordance with the intention-to-treat approach, where possible, experimental testing will be conducted for all participants who deviate from, do not adhere to, or cease participation in the intervention.

**Table 3:** Summary of experimental measures and timing of assessment

	**Assessment** **Method**	**Baseline**	**Post-Inpatient**	**12 Weeks Post-Inpatient**
**Primary Outcomes**				
-VO_2_peak	CPET	✔		✔
-Peak cardiac output	ExCMR	✔		✔
**Secondary Outcomes**				
-Quality of life	FACT-BMT	✔	✔	✔
-Fatigue	FACIT-Fatigue	✔	✔	✔
-Functional independence	CPET	✔		✔
**Exploratory Outcomes**				
-Vascular function	CMR	✔		✔
-Cardiac mass	CMR	✔		✔
-Diffuse myocardial fibrosis	CMR	✔		✔
-Diastolic and systolic cardiac function	Echocardiography	✔	✔	✔
-Thigh muscle volume	Thigh MRI	✔		✔
-Thigh muscle fat fraction	Thigh MRI	✔		✔
-Oxygen uptake kinetics	CPET	✔		✔
-Body composition	DXA	✔	✔	✔
-Cardiovascular biomarkers	Blood Samples	✔	✔	✔
-Engraftment indices	Blood Samples	✔	✔	✔
-Development of acute GVHD	Blood Samples	✔	✔	✔
-Anxiety and depression	HADS	✔	✔	✔
-Dietary intake	Diet Diary	✔	✔	✔
-Habitual Physical Activity	Accelerometry/Fitabase	✔	✔	✔
-Habitual Sedentary Time	Inclinometry/Fitabase	✔	✔	✔
-Habitual Sleep Quality	Sleep Log/Fitabase	✔	✔	✔

Cardiopulmonary exercise test (*CPET*), cardiac magnetic resonance imaging (*CMR*), exercise cardiac magnetic resonance imaging (ExCMR), functional assessment of cancer therapy with bone marrow transplant (*FACT-BMT*) questionnaire, functional assessment of chronic illness therapy (*FACIT-fatigue*) questionnaire, dual energy x-ray absorptiometry (*DXA*), graft versus host disease (*GVHD*), Hospital anxiety and depression scale (*HADS*), cardiovascular disease biomarkers include HbA1c, fasting glucose, troponin-I, BNP, LDL- and HDL-cholesterol, triglycerides

**Fig. 2:**
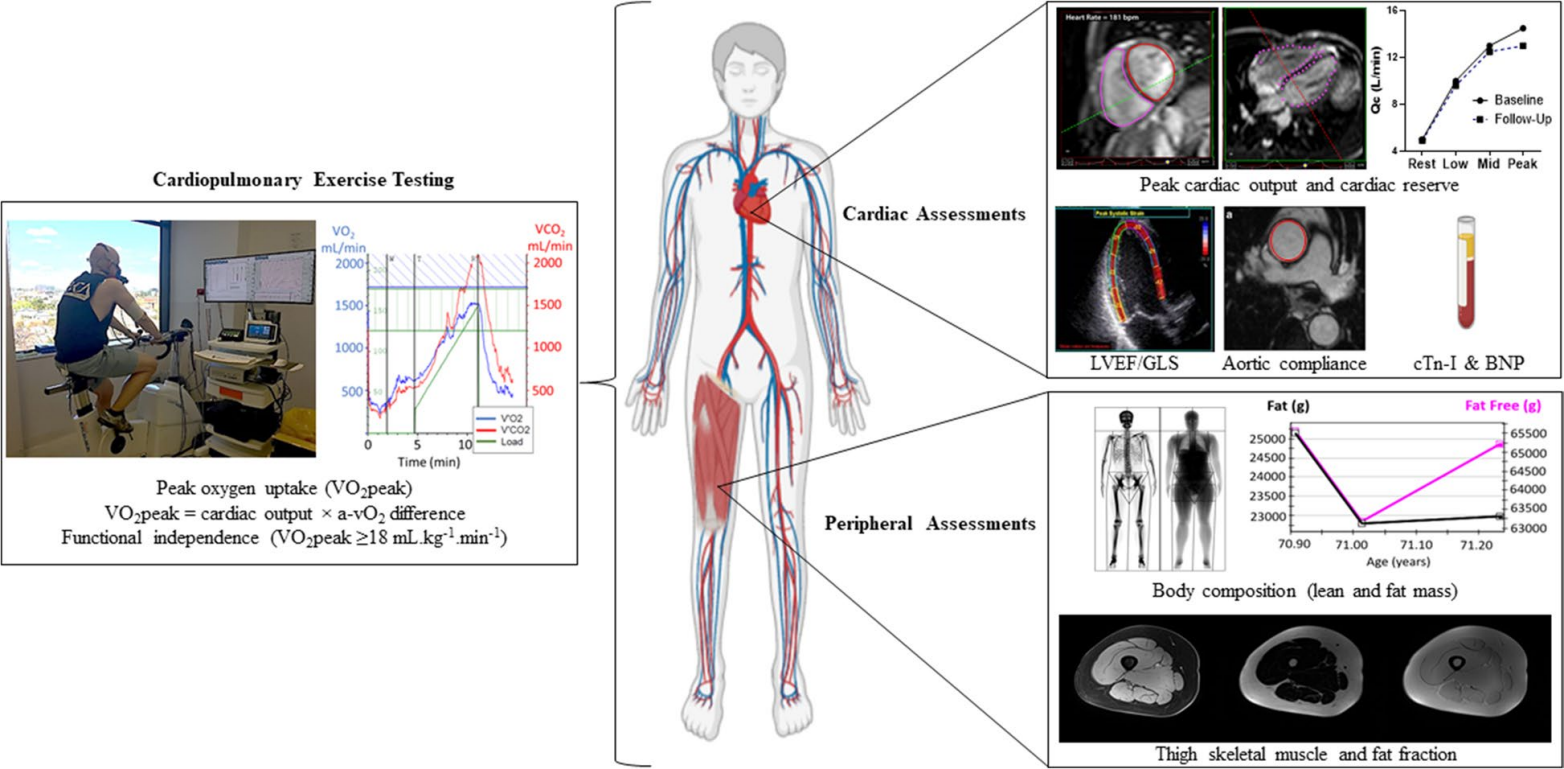
Overview of protocol assessments and outcomes. LVEF—left ventricular ejection fraction, GLS—global longitudinal strain, cTn-I – troponin-I, BNP – brain natriuretic peptide. Original figure – individual image components sourced from testing at our laboratory

The primary outcomes for assessing the efficacy of the intervention on cardiovascular health will be change in VO_2_peak assessed via cardiopulmonary exercise testing and peak cardiac output assessed via exercise cardiac magnetic resonance imaging (ExCMR) (multiple primary outcomes). Secondary outcomes will include prevalence of functional independence (VO_2_peak ≥ 18 mL.kg^−1^.min^−1^) as well as patient reported fatigue and quality of life. Exploratory outcome measures will include changes in cardiac reserve, indices of resting cardiac structure and function (cardiac mass, myocardial fibrosis, diastolic and systolic function), central vascular compliance, cardiovascular biomarkers (troponin-I [cTn-I], brain natriuretic peptide [BNP], glycaemic control, lipid profile and adiposity), skeletal muscle structure and function (lean body mass, thigh muscle cross-sectional area and fat fraction, oxygen uptake kinetics, and muscular strength, power and stability) as well as anxiety, depression and transplant related outcomes including engraftment indices (time to neutrophil and platelet engraftment, immune reconstitution) and development of GVHD. The study will also assess habitual physical activity and dietary intake due to their potential impact on cardiovascular outcome measures. An overview of the protocol assessments and outcomes can be seen in Fig. 2.

### Cardiopulmonary fitness

Cardiopulmonary exercise testing (CPET) will be used to assess cardiorespiratory fitness (VO_2_peak). This will be performed on an electronically braked cycle ergometer (Lode Excalibur Sport, Lode BV Medical Technology, Groningen, NL) with breath-by-breath expired gas analysis (Vyntus™ CPX, CareFusion, San Diego, CA). Heart rate, rhythm and blood oxygen saturation will be monitored continuously with a 12-lead ECG (Vyntus CPX, Care Fusion, San Diego, CA) and ear clip pulse oximeter, respectively. Brachial blood pressure will be measured at 2 min intervals (Tango M2 ECG-gated automated blood pressure monitor, SunTech Medical Inc., Morrisville, NC). Two minutes of resting data will be collected prior to commencing exercise, following which, an unloaded steady state exercise test will be conducted to assess skeletal muscle oxygen kinetics [62]. Following the unloaded cycling (~ 3–5 min) participants will commence a continuous ramp protocol (5–25 W/min), individualised to participant’s age, weight, and self-reported physical activity levels. Blood lactate concentrations will be assessed at rest, when respiratory exchange rate = 1 (RER1), peak exercise, and 2 min post-exercise via finger prick blood sample (Lactate Pro2, Arkray, Japan). Two minutes of recovery data will be collected following CPET completion to assess recovery oxygen kinetics. Standard criteria will be applied for the assessment of achieving VO_2_peak including the attainment of at least two of the following: volitional fatigue, > 85% age predicted heart rate maximum, RER > 1.1, or blood lactate concentration > 8.0 mmol/L [63]. VO_2_peak will be defined as the average of the six consecutive highest 5 s VO_2_ values, with functional independence classified as VO_2_peak ≥ 18 mL.kg^−1^.min^−1^. Ventilatory threshold (VT) will be assessed using the V-slope method, and the relative proportion of VO_2_peak at which the VT occurs will be used as a measure of changes in submaximal exercise capacity. Ventilatory efficiency (V_E_/VCO_2_ slope) will be obtained via linear regression analysis of minute ventilation (V_E_) and expired carbon dioxide (VCO_2_) from the end of the warm-up to the VT.

### Cardiac reserve

#### Exercise cardiac magnetic resonance imaging

The biventricular response to supine cycle exercise will be evaluated using a real-time CMR protocol that has been detailed previously and validated against invasive measures [64]. In brief, ungated real-time steady-state free-precession (SSFP) cine MR imaging will be conducted using a Siemens MAGNETOM Prisma 3.0 T CMR with a five-element phased array coil. Forty to 75 consecutive frames will be acquired at a temporal resolution of 36–38 ms for each ~ 10–18 adjoining 8 mm image slices in both the short and long-axis planes, encapsulating both ventricles. Imaging parameters will be as follows: field of view, ~ 320 × 260 mm; matrix, 128 × 128; flip angle, 50°; SENSE factor, 2 (Cartesian k-space undersampling); repetition time, 1.8 for ms; echo time, 0.9 ms; and reconstructed voxel size, 2.3 × 2.3 × 8 mm. Cardiac images will be obtained at rest, and whilst cycling on an MR compatible supine cycle ergometer (MR Ergometer Pedal, Lode, Groningen, the Netherlands) at workloads corresponding to 20%, 40%, and 60% of the maximal power output achieved during upright CPET as this approximates low, moderate, and maximal exercise capacity in the supine position [64, 65]. Each workload will be maintained for 1.5–3 min (comprising ~ 30 s to achieve a physiological steady state, and 1–2.5 min for image acquisition), with the imaging duration decreasing with each increasing intensity level.

Real-time cine images will be analysed offline in a software developed in-house (Right Volume Software Program, Leuven, Belgium). Images will be retrospectively synchronised to cardiac and respiratory cycles to ensure endocardial contouring is performed at end-expiration for consistency. The left and right ventricular endocardial contours will be manually traced in the short-axis plane, and the transection points with the long-axis plane will be marked, to allow cross-referencing to the atrioventricular valve plane when defining endocardial contours. Ventricular volumes will be quantified at rest and at each increasing exercise intensity level (low, moderate, and high) via the summation of disc method (papillary muscles and trabeculations included in the blood pool). Stroke volume index (SVI) will be obtained by subtracting end-systolic volume (RVESV + LVESV/2) from end-diastolic volume (RVEDV + LVEDV/2) indexed to body surface area (BSA). Cardiac index (CI) will be calculated by multiplying SV (RVSV + LVSV/2) by HR indexed to BSA. Left and right ventricular ejection fractions (LVEF, RVEF) will be calculated as the ratio of EDV to SV, multiplied by 100 to be reported as a percentage. Cardiac (CI, SVI, HR) and contractile (LVEF, RVEF) reserve will be defined as the heart's ability to augment its function in response to maximal exercise (peak exercise value – rest value). Using this novel technique of cardiac volume analysis, our laboratory has demonstrated exceptional inter-study and inter-observer reproducibility (inter-study cardiac output: *R* = 0.98; inter-observer left- and right-ventricular SV: *R* = 0.98 and *R* = 0.97, respectively) [64].

### Resting cardiac structure and function

#### Resting echocardiography

A comprehensive resting echocardiogram assessment will be used to characterise cardiac structure and function (Vivid E95, GE Healthcare, Horten, Norway). This is the current standard of clinical care and will include Doppler and 3-dimensional volumetric acquisitions and torsion, global longitudinal strain (GLS), and strain rate measures. Left-ventricular ejection fraction (LVEF) will be quantified and used to assess left ventricular systolic function according to standard recommendations. All images acquired will be analysed using offline analysis software (Echopac V13.0.00, GE, Norway).

### Cardiac magnetic resonance imaging

Using an identical protocol as described previously [66], myocardial tissue characterisation and cardiac mass will be assessed using non-contrast T1 mapping and breath-hold SSFP sequences, respectively.

### Vascular function

#### Blood pressure

Supine systolic- and diastolic blood pressure will be measured from the brachial artery using an oscillometric blood pressure monitor (OMRON HEM-907, OMRON Corporation, Tokyo, Japan). Measurements will be taken after ≥ 10 min rest in the supine position and the average of three measurements, taken ≥ 3 min apart, will be used for analysis. Where one of three measurements is substantially different from the other two, a fourth measurement will be taken.

#### Cardiac magnetic resonance imaging

Central (aortic) compliance will be assessed using ECG-gated resting CMR cine-imaging conducted prior to the ExCMR, as a measure of vascular function. Transverse images of the ascending aorta will be taken just above the sinotubular junction. Cine images will be analysed for changes in 2-dimensional area across the cardiac cycle that can be incorporated with SV (calculated from breath-hold SSFP images) and pulse pressure (obtained from brachial blood pressure measured by an automated cuff) to calculate aortic distensibility and compliance in line with previously validated methods [67].

### Anthropometrics, body composition and bone mineral density

#### Anthropometry

Body height and weight will be measured using a fixed stadiometer (SECA, Hamburg, Germany) and portable scales (Coverall Medical Technologies, Victoria, Australia), and used to calculate body mass index and BSA.

#### Dual energy X-ray absorptiometry

Total body and regional lean mass, fat mass and percentage body fat will be measured via a total body dual energy X-ray absorptiometry (DXA) scan (GE Lunar iDXA, GE Healthcare, Little Chalfont, United Kingdom). DXA has the capacity to efficiently and non-invasively measure total body composition with excellent precision and accuracy (coefficient of variation < 1%) [68, 69]. Scans will be manually analysed using enCore analysis software (version 14.10.022) in line with standardised procedures.

#### Magnetic resonance imaging

Quadricep muscle cross-sectional area and intramuscular fat content will be assessed by two-point Dixon-based MRI (Siemans Prisma 3 T MRI). The two-point Dixon method has been validated as an accurate and reproducible (CV = 0.6%) measurement of muscle-fat content. MRI scans of the right thigh will be acquired in the supine position, from the superior patella to the greater trochanter. Images will be analysed using openly available ImageJ software (ImageJ2 v1.52d) to manually outline the regions of interest (rectus femoris, vastus intermedius, vastus medialis and vastus lateralis). Muscle volume will be calculated from the summation of disks method by multiplying the sum of the combined regions of interest by the inter-slice distance. Fat fraction will be calculated from the ratio between the fat and combined fat and water signal intensities for the regions of interest.

### Biochemistry

Fasting blood samples will be collected. Analysis of these samples will be performed by the Alfred Hospital Pathology Laboratory. The assessments performed will include a full blood examination, liver function tests, and serum chemistry. In addition, markers of myocardial injury (cTn-I) and myocardial stress (BNP), glycaemic control (fasting glucose, HbA1C), and lipid profiles (total-, LDL- and HDL cholesterol and triglycerides) will be collected.

### Physical function

Physical function testing will be performed as per standardised guidelines and include the six-minute walk test, hand-grip strength test and the short physical performance battery (SPPB) encompassing the five times sit-to-stand test, 4 m usual gait speed test, and static balance measured by side-by-side, semi-tandem and tandem stand balance tests [70, 71]. SPPB scores range from zero (high frailty) to 12 (low frailty) possible points. A score of ≥ 10 will be considered ‘normal’, while scores of 7–9, 4–6 and 0–3 will indicate mild, moderate and severe limitations in lower extremity function, respectively [72].

### Quality of life, fatigue, anxiety and depression

Quality of life will be assessed using the Functional Assessment of Cancer Therapy with Bone Marrow Transplant (FACT-BMT)—a 50 item subscale specifically designed to assess multidimensional quality of life in bone marrow transplant recipients. Fatigue will be assessed using the 13 item Functional Assessment of Chronic Illness Therapy – Fatigue subscale (FACIT-Fatigue), while anxiety and depression will be assessed using the Hospital Anxiety and Depression Scale (HADS). All questionnaires have been validated in cancer populations.

### Diet, physical activity, sedentary time, and sleep

#### 3 day food diary

Habitual dietary intake will be assessed using a 3 day diet record completed using the Easy Diet Diary smartphone application. Participants will be required to record the type and quantity of food and beverage consumed over a 24 h period for two weekdays and one weekend day. Data entered into Easy Diet Diary will be exported into FoodWorks 10 Professional (Xyris, Australia), and average energy intake and macronutrient composition will be quantified.

#### 7 day accelerometry and inclinometry

Habitual physical activity and sedentary time will be assessed over 7 days utilising a hip-worn accelerometer (Actigraph GT3X +) and thigh-worn inclinometer (ActivPAL3). This wear-time aligns with recommendation for objective physical activity assessment in cancer cohorts. Inclinometer data will be exported using ActivPAL3™ software (v.8.10.9.46, PAL Technology, UK) and analysed for time spent sedentary (h/day), in prolonged sitting ≥ 60 min (h/day), standing (h/day), and stepping (h/day). Actigraph data will be exported using ActiLife software (ActiGraph, V6.13) and used to assess energy expenditure (metabolic equivalent of task [MET] min/day) and daily activity levels based on Freedson cut-points for sedentary, light, moderate and vigorous physical activity levels [73].

#### 7 day sleep log

Sleep quantity and quality will be assessed using a 7 day sleep log. Participants will document the time of sleep onset and cessation, the frequency and length of any awakenings and rate their sleep quality on a 5-point scale from very poor to very good.

#### Fitabase

Additional information regarding habitual physical activity, sedentary behaviour and sleep will be collected via Fitbit devices and collated on Fitabase (a cloud-based data aggregation platform). Specifically, Fitbit devices will be worn continuously for the entirety of the study (~ 4 months) to allow comprehensive longitudinal insight into the physical activity (e.g., number of steps [steps/day], as well time spent in light, moderate and high intensity activity [min/day]), sedentary behaviour (e.g., time spent sitting [min/day], time spent in prolonged sitting [≥ 60 min/day], and sleep quality and quantity (time spent awake, and in light, REM, or deep sleep [min/day]) of participants.

### Transplant outcomes

Additional transplant-related outcomes will be recorded from Alfred Health patient medical records including the duration of hospital stay during the transplant procedure, time to engraftment, GVHD prophylaxis, development and severity, steroid use, and other relevant medications and/or complications (including rehospitalisation).

### Semi-structured interview (Ex + SED group only)

A semi-structured interview with an interpretive phenomenological framework will be conducted to evaluate the lived experiences of those who participated in the ALLO-Active Ex + SED Program. The interview will be held in a private room, audio recorded, and follow a semi-structured interview guide designed to extract information relating to barriers, facilitators, positives and negatives, and overall experience in the ALLO-Active Ex + SED Program.

### Ex + SED adherence and attendance

Attendance and adherence to the prescribed structured exercise sessions, and exercise dose within each exercise session will be manually documented into a REDCap (REDCap, Vanderbilt University, Nashville, USA) database. Attendance will be calculated as the number of exercise sessions attended versus prescribed. Adherence to the prescribed exercise dose will be calculated as the duration and intensity (aerobic component) or number of exercises, sets, repetitions, and weight (resistance component) completed vs prescribed. The supervising exercise professional will document the reason for each scheduled supervised exercise session that is not completed (i.e., clinical contraindication or self-selection) and for any modifications made to the prescribed aerobic or resistance exercise. For unsupervised home-based aerobic sessions (Phase 2), participants will be provided a hard copy aerobic based program, on which they will document the duration and intensity of their exercise completed. Research staff will then cross-check the returned programs with participants’ Fitabase data to determine the level of adherence to the prescribed duration and intensity. Adherence to the hourly unsupervised sedentary behaviour breaks will be automatically documented into Fitabase (Fitabase Small Steps Labs, LLC, San Diego, CA) upon syncing of each participant's Fitbit data, and calculated as the average number of hourly sedentary behaviour breaks completed per day versus prescribed.

### Data management

A unique study code will be assigned to all participants to ensure the confidentiality of their data is maintained. The secure online data management system REDCap (REDCap, Vanderbilt University, Nashville, USA) will be utilised to store all data and this will only be accessible to members of the study team. All data entered electronically will be double-checked for accuracy. Due to the low risks associated with the exercise components of the intervention, there will not be a formal data monitoring committee for this study. However, the study team will meet monthly to review study progress and data will be checked at regular intervals throughout the study.

### Sample size calculation

No prior studies have directly assessed the effect of a structured exercise intervention on changes in VO_2_peak and cardiac output in this patient cohort. As such, we based our sample size calculations on data collected from our group’s previously published work that utilised a similar exercise intervention in women being treated for breast cancer [74], whereby clinically meaningful changes in cardiorespiratory fitness and cardiac function were observed. The present study is powered to detect a minimum between-group difference in VO_2_peak of 10% (SD = 11%, effect size = 0.91) and cardiac output of 9% (SD = 10%, effect size = 0.9) after treatment, assuming a conservative correlation coefficient of *r* = 0.4 between baseline and follow-up testing. To detect this difference 20 completions per group are required (80% power; alpha = 0.025; multiple primary endpoints). To account for withdrawals, mortality associated with disease progression and treatment, and uncertainty related to the COVID-19 pandemic, a total of 60 participants will be recruited (Ex + SED; *n* = 30 and UC; *n* = 30).

### Statistical analyses

To examine the differential effects of the intervention on VO_2_peak, peak cardiac output, and additional secondary and exploratory outcome measures, generalised linear mixed modelling will be conducted (adjusted for baseline values) with participants as the random effect, time as the repeated measures factor, and group and group-by-time interactions as the fixed effects. The covariance structure of the model will be informed by the Akaike information criteria. The primary analysis will be conducted on an intention-to-treat basis and a probability level of 0.025 will be adopted to account for multiple primary outcome endpoints. Standardised effect sizes (Cohen’s *d*) will be calculated to determine the magnitude of change for within and between group effects. A per protocol analysis including participants who completed > 66% of the planned exercise sessions will also be performed.

### Adverse events

The occurrence of any adverse events directly related to the procedures involved in this protocol will be collected during all testing visits. Participants randomised to the Ex + SED group will also be asked about the occurrence of adverse events resulting from the intervention prior to each exercise training session. Any untoward medical occurrence in a participant that may have a causal relationship with the intervention will be reported as an adverse event. A serious adverse event will be defined as any life-threatening, hospitalisation, disability or death that occurs because of an incident within two hours of completing an exercise session. Hospitalisations and deaths related to disease progression will not be reported as adverse events given the complex treatment and complications that are often associated with allo-SCT.

### Dissemination

The study results will be presentated at medical and scientific conferences and published in a peer-reviewed clinical journal. The results of this study will also help meet the requirements of research higher degree completions. Participants will receive a lay summary of the study results.

### Participant withdrawals

A participant may withdraw from the study at any time. All data that has been collected from the participant up until the point of withdrawal will be included in the final analyses (unless withdrawal occurs prior to randomisation). Participants that are lost-to-follow-up, decline to complete testing (due to perceived incapacity, injury, or changes in personal circumstances), or who are deceased will not be deemed as withdrawals from the study and all data collected from these participants will be included in the final analyses.

## Discussion

Allo-SCT survivors experience elevated rates of premature cardiovascular disease [6, 13–18], functional impairment and poor quality of life [9, 11, 12, 75]. Mounting evidence from observational studies indicates these cardiovascular impairments are apparent within the early stages of the transplant process [11, 12], suggesting that this may be an opportune time to intervene and mitigate these consequences. Exercise interventions have been shown to prevent functional decline in oncology populations and may also be an efficacious primary preventative approach for allo-SCT patients [76]. This study will comprehensively evaluate the efficacy of intervening early in the transplant process with a multi-faceted exercise intervention in mitigating the detrimental effects associated with allo-SCT.

Currently, the utility of exercise as a potential therapeutic strategy to counteract the detrimental cardiovascular consequences associated with allo-SCT is not well characterized. While previous studies have demonstrated a beneficial effect of exercise on functional capacity in allo-SCT recipients [11, 31, 33, 35–39], these studies have varied significantly in the type, timing and duration of intervention delivered, as well as the cardiovascular endpoints assessed. Indeed, in contrast to previous studies, the ALLO-Active trial will incorporate strategies to not only increase purposeful aerobic and resistance exercise but also reduce and break-up sedentary time through replacement with light-intensity exercise. This novel multi-faceted exercise approach—which was informed by evidence that prolonged sedentary behaviour poses a detrimental risk to cardiovascular health independent of physical activity levels [43, 44]—has the potential to benefit cardiovascular outcomes in allo-SCT recipients. Specifically, we hypothesise that this will occur through a combination of systemic and molecular mechanisms that act to oppose the negative effects of physical deconditioning, cancer therapies and allografting on metabolic and growth signalling pathways in the heart and periphery. Furthermore, the ALLO-Active intervention will be delivered during allo-SCT and acute inpatient recovery (~ 4 weeks) and for 12 weeks post-discharge (~ 16 weeks total). This timing is designed to prevent the cardiovascular consequences as they occur throughout the transplant process. While some studies have intervened at this early stage [11, 31, 32, 35, 77], the absence of comprehensive cardiovascular evaluation and insufficient intervention durations (4–10 weeks) mean that conclusive evidence supporting the therapeutic utility of exercise and sedentary behaviour reduction (through replacement with light exercise) as a primary preventative strategy is still required. Indeed, the ALLO-Active trial will be the first to quantify the efficacy of a multi-faceted exercise intervention (exercise combined with interruption in sedentary time) in preserving directly measured VO_2_peak (via gold standard cardiopulmonary exercise testing). VO_2_peak encapsulates the integrative functioning of the cardiovascular, hematological, and skeletal muscle systems, has proven prognostic utility for predicting cardiovascular risk and outcomes, and therefore, is likely to be sensitive to the beneficial effects of the intervention. The ALLO-Active trial will also be the first to comprehensively characterise the effect of an exercise and sedentary behaviour intervention on the cardiac, vascular, hematological, and skeletal muscle factors that underlie changes in VO_2_peak and cardiovascular risk following allo-SCT. This will include novel resting echocardiographic and ExCMR imaging methods, as well as aortic and thigh MRI which are sensitive to subclinical changes in cardiac, vascular, and skeletal muscle, respectively. Taken together, this trial will provide important information regarding the efficacy of exercise in counteracting the multiple cardiovascular insults imposed by allo-SCT.

### Practical implications

Currently, guidelines for patients undergoing cancer treatment and for cancer survivors support the incorporation of exercise as a part of standard care and recommend adherence to typical physical activity and sedentary behaviour guidelines for the general population [78, 79]. Exercise has been shown to be safe and effective for reducing the burden of many adverse physical and psychological effects associated with cancer treatment [80–83]. However, optimising the type and timing of specific exercise interventions in allo-SCT patients requires further investigation. In this regard, the outcomes from this study will help to inform future evidence-based guidelines for allo-SCT patients for clinicians and provide insight regarding the efficacy of an exercise intervention for mitigating the detrimental cardiovascular effects and reductions in quality of life associated with the treatment. The feasibility and adherence to the intervention will be evaluated once the study has been completed to assist with the design of future, larger cohort, and multicentre studies.

### Practical challenges, strengths, and limitations

There are challenges associated with conducting this type of intervention within a clinical cohort that commonly experience a wide range of treatment-related adverse effects. From a practical perspective, the ability of the patients to perform the intervention in accordance with the study protocol may be impacted by the erratic health status of the individuals throughout their treatment. Indeed, in this acute setting, it is common for allo-SCT patients to report high levels of nausea, fatigue, breathlessness, muscle pain, diarrhea, and loss of appetite, amongst many other side effects [84]. Furthermore, low hemoglobin and platelet levels are inherent to allo-SCT and can persist throughout acute inpatient and outpatient recovery. As such, all exercise sessions will be individualised to accommodate each participant’s current health status. For instance, both objective (heart rate and workload) and subjective (RPE) measures of intensity will be assessed to accommodate any daily fluctuations in physiological presentation.

Despite these challenges, the study has several strengths. These include gold standard CPET assessments of VO_2_peak combined with comprehensive cardiovascular and peripheral imaging techniques, which will provide a detailed characterisation of the effect of allo-SCT (and therapeutic benefit of the intervention) on VO_2_peak and the determinants underlying changes in VO_2_peak and cardiovascular risk in this population. Moreover, this type of intervention which has low barriers for initiation and high potential for continuation may be particularly accessible in this cohort. Limitations associated with our study design including participant recruitment from a single site and a relatively small sample size. The nature of a study involving an exercise intervention may also be biased towards the recruitment of participants who are already physically active; therefore, the usual care group may reflect a population more motivated and physically active than typically observed in this cohort. The lack of blinding for the assignment of a participant’s study group, although unavoidable, may also be considered a study limitation.

In summary, the ALLO-Active study will be the first randomised, controlled trial to examine the efficacy of intervening early with a multi-faceted exercise program to preserve cardiovascular function in allo-SCT recipients. The primary outcomes will focus on changes in VO_2_peak and peak cardiac function, while the secondary aims will evaluate the efficacy of the intervention to improve quality of life, and reduce fatigue and prevalence of functional disability (VO_2_peak < 18 ml.kg^−1^.min^−1^). The outcomes of this study will assist with the development of evidence-based guidelines for recommending exercise as an adjunct therapy throughout allo-SCT.

### Trial status

At the time of submission, participants are currently being recruited and enrolled.

## Data Availability

The datasets used and/or analysed during the current study are available from the corresponding author on reasonable request.

## Abbreviations

ACSM: American College of Sports Medicine
Allo-SCT: Allogeneic stem cell transplant
AT: Aerobic training
BHDI: Baker Heart and Diabetes Institute
BNP: Brain natriuretic peptide
BSA: Body surface area
CI: Cardiac index
CMR: Cardiac magnetic resonance imaging
CPET: Cardiopulmonary exercise test
cTn-I: Troponin-I
CVD: Cardiovascular disease
DXA: Dual energy X-ray Absorptiometry
EDV: End-diastolic volume
ESV: End-systolic volume
Ex + SED: Exercise and sedentary behaviour intervention
ExCMR: Exercise cardiac magnetic resonance imaging
FACIT-Fatigue: Functional Assessment of Chronic Illness Therapy – Fatigue subscale
FACT-BMT: Functional Assessment of Cancer Therapy with Bone Marrow Transplant
GLS: Global longitudinal strain
HADS: Hospital Anxiety and Depression Scale
HR: Heart rate
HR_peak_: Heart rate peak
LV: Left ventricular
LVEF: Left ventricular ejection fraction
MET: Metabolic equivalent of task
MRI: Magnetic resonance imaging
RER: Respiratory exchange ratio
RPE: Rating of perceived exertion
RT: Resistance training
RV: Right ventricular
RVEF: Right ventricular ejection fraction
SPPB: Short physical performance battery
SSFP: Steady-state free precession
SVI: Stroke volume index
UC: Usual Care
VCO_2_: Volume of carbon dioxide production
V_E_/VCO_2_ slope: Minute ventilation to carbon dioxide production slope
V_E_: Minute ventilation
VO_2peak_: Volume of peak oxygen consumption
VT: Ventilatory threshold
